# Phylogenetic Analysis of Hepatitis E Virus in Northwest India

**DOI:** 10.1155/2012/976434

**Published:** 2012-10-24

**Authors:** Nidhi Subhash Chandra, Ramesh Roop Rai, Bharti Malhotra

**Affiliations:** ^1^Advanced Research Lab, SMS Medical College, Jaipur, Rajasthan 302004, India; ^2^Gastroenterology Department, Fortis Escorts Hospital, Jawaharlal Nehru Marg, Malviya Nagar, Jaipur, Rajasthan 302017, India

## Abstract

Genotyping and subtyping are important to understand epidemiology of the hepatitis E virus so as to improve control measures to prevent transmission of virus in the community. Hence, the aim of the current study was to identify the prevalent HEV genotypes in Rajasthan in acute sporadic hepatitis E cases with varying degree of liver failure. We studied hepatitis E virus (HEV) isolates from hospitalized patients in Rajasthan, western India. In a total of seventeen HEV sequences, six acute viral hepatitis, seven acute liver failure, and 4 acute- on-chronic cases were analyzed. Subtypes 1a and 1c of HEV are prevalent in Northwest India.

## 1. Introduction 

Hepatitis E is an enterically transmitted disease that spreads through faecal contamination of drinking water. It occurs both in the form of epidemics as well as sporadic infection in developing countries [1, 2]. It is endemic to the Indian subcontinent, where the seroprevalence rate ranges between 4 and 20%. More than 60% of acute viral hepatitis cases are attributed to HEV [3]. Hepatitis E virus affects young to middle aged adults and causes high mortality in pregnant women, 20–30% as compared to 0.2–1% in general population [3]. It has been implicated as an important aetiological agent for sporadic fulminant hepatic failure (FHF) in developing countries [4].

HEV belongs to the genus *Hepevirus* in the family Hepeviridae and has a 7.2 kb positive-sense single-stranded RNA genome [5]. The HEV genome has three open-reading frames (ORFs). The ORF1, ORF2, and ORF3 encode nonstructural proteins including an RNA-dependent RNA polymerase (RdRp), a capsid protein, and a small protein that possibly induces immune suppression in HEV-infected patients, respectively [6, 7]. Presently, HEV is classified into four major genotypes [8]. Further classification of genotypes into various subtypes was given by Lu et al., 2006 [9]. Genotypes 1 and 2 have been identified exclusively in humans, and genotypes 3 and 4 have been found in humans and several animal species. Genotypes 1 and 2 have been isolated in Asia, Africa, North America; genotype 4 has been identified only in Asia; and genotype 3 has been found in almost every country [10].

All parts of India have been experiencing repeated outbreaks and sporadic cases of HEV since 1955 [11–14] with genotype 1 being prevalent in the human population. With the development of knowledge about the circulation of subtypes of HEV, this study is aimed at the molecular characterization of HEV isolates to determine the most prevalent genotype in Northwest India (Rajasthan).

## 2. Materials and Methods

### 2.1. Patient

The present study was carried out on 585 acute hepatitis patients attending the OPD or admitted in wards of Gastroenterology Department of the SMS Medical College and Hospital Jaipur, Rajasthan a tertiary care centre, from September 2006 to December 2009. The study was approved by the institutional ethics committee and informed written consent was taken from the patients. On the basis of disease severity the study comprised three HEV-induced groups: acute viral hepatitis (AVH), acute liver failure (ALF), and acute-on-chronic liver disease (ACLF), respectively. The diagnostic criterion for AVH, ALF, and ACLF was as follows.

AVH is marked by appearance of jaundice with or without prodrome and raised ALT and AST levels. 

ALF is defined as the rapid development of hepatocellular dysfunction, specifically coagulopathy and mental status changes (encephalopathy) in a patient without known prior liver disease and with an illness of <24 weeks duration. 

ACLF is defined by the Asian Pacific Association for the Study of Liver (APASL, 2008) as an acute hepatic insult manifesting as jaundice and coagulopathy, complicated within 4 weeks by ascites and/or encephalopathy in a patient with previously diagnosed or undiagnosed chronic liver disease (CLD). 

Patients with stable compensated chronic liver disease, significant comorbid illnesses like coronary artery disease, renal failure, and cerebrovascular disease were excluded from the study. 

### 2.2. Sample Collection

10 mL blood samples were collected from all the cases. The serum was separated and stored at −80°C taking precaution avoiding repeated freezing and thawing of the samples by aliquating them separately for serology and PCR. The clinical symptoms of the patients were recorded simultaneously and all the biochemical tests were performed. 

### 2.3. Serology

Serum samples were screened using commercially available Micro-ELISA for markers of hepatitis E (EIAgen HEV IgM, Adaltis, Spain). The kit was coated with recombinant proteins for open-reading frames (ORFs) 1 and 2 with 98% sensitivity and specificity. ELISA was performed as per manufacturers' protocol. 

### 2.4. Biochemical Profile

The following biochemical parameters were done for all the patients: (i) serum alanine aminotransferase (ALT), (ii) serum aspartate aminotransferase (AST), (iii) alkaline phosphatase (ALP), and (iv) total bilirubin (TB).

### 2.5. RNA Extraction

RNA extraction from serum of acute hepatitis E cases was done by GITC chloroform phenol method with minor modification [15].

### 2.6. RT-PCR

Extracted RNA was subjected for cDNA synthesis. cDNA synthesis was carried out using MuLV RT enzyme, reverse primer (20 pmol/mL) (external antisense: 5′-CCG AAT TCA AAG GCA TCC ATG GTG TTT GAG AAT GAC- 3′) (Promega), RNase out (20 U/*μ*L, invitrogen), 0.1 M DTT, and 5 *μ*L templates at 42°C for one hour. 

After cDNA synthesis PCR amplification was carried out using the specific previously validated primers selected from nonstructural ORF1 region (Gene Bank accession no. M-32400) [1]. The primers used were external sense: 5′- CCG GAT CCA CAC ACA TCT GAG CTA CAT TCG TGA GCT- 3′, external anti-sense: 5′- CCG AAT TCA AAG GCA TCC ATG GTG TTT GAG AAT GAC- 3′, internal sense: 5′- GGA ATT CGA CTC CAC CCA GAA TTA CTT- 3′, and internal anti-sense 5′- GGA ATT CAC AGC CGG CGA TCA GGA CAG- 3′. These two sets of primers were designed to produce 343 bp segment of ORF1 region [1].

The thermal cycling conditions were as follows: initial denaturation 94°C for 5 minutes followed by 30 cycles of denaturation for 30 seconds at 94°C, annealing for 30 seconds at 59°C, and extension for 30 seconds at 72°C, as well as final extension for 7 minutes at 72°C. Positive and negative control was included in every reaction to rule out false positive and negative. 

### 2.7. DNA Sequencing

Amplified final PCR products (343 bp of RdRp region) were separated in a 1.5% gel. The expected bands were excised from the gel and purified with a QIA quick gel extraction kit (Qiagen, Hilden, Germany). The purified DNA were subjected to sequencing PCR using big dye terminator ready reaction mixture, reverse primer (3.2 pmoles/*μ*L) at thermal cycling conditions: 25 cycles of denaturation 94°C for 10 seconds, annealing 55°C for 5 seconds and extension 60°C for 4 minutes. Cycle sequencing PCR product was purified by adding 0.1 volume of 3 M sodium acetate (pH = 4.5) and 2.5 volume of absolute alcohol and dissolved in 25 *μ*L of template suspension reagent (TSR) and heated at 95°C for 5 minutes and snap-chilled to denature the DNA. Samples were transferred in fresh tubes, and closed with septa. These samples were loaded on sample tray. The sample was run through performance-optimized polymer (POP6) and electrophoreses at 12.1 kv for 3 hours in 1 Xgenetic analyser buffer on ABI prism 310 sequencer.

### 2.8. Phylogenetic Analysis

Amplicon sequences of HEV RdRp region were compared to an online database for the best possible match using the BLAST (Basic Local Alignment Search Tool) program of National center for Biotechnology information (http://www.ncbi.nlm.nih.gov/) and CLUSTAL-X version 2.0. The evolutionary history was inferred using the Neighbor-Joining method [16]. The optimal tree with the sum of branch length = 1.92628740 is shown. The percentage of replicate trees in which the associated taxa clustered together in the bootstrap test (1000 replicates) is shown next to the branches [17]. The tree is drawn to scale, with branch lengths in the same units as those of the evolutionary distances used to infer the phylogenetic tree. The evolutionary distances were computed using the Kimura 2-parameter method [18] and are in the units of the number of base substitutions per site. Codon positions included were 1st + 2nd + 3rd + noncoding. All positions with less than 95% site coverage were eliminated, that is, fewer than 5% alignment gaps; missing data and ambiguous bases were allowed at any position. There were a total of 189 positions in the final dataset. Phylogenetic analyses were conducted in MEGA4 [19]. 

Subgenomic sequences of known HEV genotypes were retrieved from NCBI database (http://www.ncbi.nlm.nih.gov/nuccore/). Only those sequences, which had our target region of HEV RdRp, were taken for phylogenetic analysis.

## 3. Statistical Analysis

For data management and statistical analysis, SPSS-10 software (SPSS Inc., Chicago, IL, USA) was used. Baseline laboratory markers were expressed as mean values with standard deviation. Difference between AVH, ALF, and ACLF with respect to various liver function tests was calculated using the ANOVA (analysis of variance). *P* value of less than 0.05 was considered significant.

## 4. Results

In the study, a total of 585 acute hepatitis cases were included. Amongst these 205 (35.04%) were found to be positive for IgM anti-HEV antibodies. These were grouped into 100 patients with acute viral hepatitis (AVH), 75 with acute liver failure (ALF), and 30 with acute on chronic liver failure (ACLF). Age presentation, sex distribution, and characteristic features of different groups were as shown in Table 1. Maximum numbers of cases of HEV were seen in the age group 15–40 years. Out of 67 consecutive females, 27 (40.16%) were pregnant. The mortality rate in pregnant females was 6/27 (22.22%) and in nonpregnant females was 1/40 (2.5%). There was no mortality in 100 patients of AVH, while 9/75 (12%) ALF patients and 2/30 (6.67%) SAHF patients died.

Variation was observed in the different groups with respect to clinical symptoms like fever, pruritus, anorexia, and pedal edema as shown in Table 2. The age and LFT profile (AST, ALT, and ALP) was significantly higher in ALF cases than SAHF and AVH but no significant difference was obtained in total serum bilirubin as shown in Table 3. 

Due to the transient viremic nature of HEV infection, 80/205 samples were selected (within 1 week of the illness) for HEV RNA detection by nested reverse transcriptase polymerase chain reaction (nRT-PCR). 47/80 (58.75%) were positive for HEV-RNA. Only 17 from different groups were selected for sequence analysis. 

 The amplification products of RdRp region of ORF1 (343 nt) from 6 AVH patients, 7 ALF patients, and 4 acute on chronic patients were subjected for sequence analysis and submitted to GenBank with accession nos. FJ231471, FJ231472, FJ231473, FJ231474, FJ231475, FJ231476, FJ231477, FJ231478, FJ231479, FJ231480, FJ231481, FJ231482, FJ231483, FJ231484, FJ231485, FJ231486, FJ231487. Gene sequences of the present isolates along with standard strains from different geographical areas were used for the construction of the phylogenetic tree.

**Figure 1:**
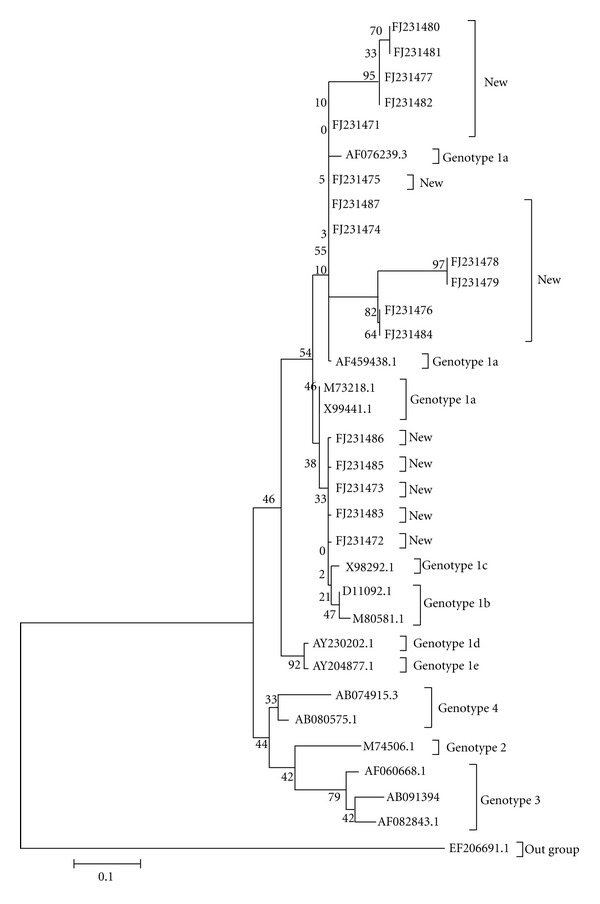
Molecular phylogenetic analysis by neighbor-joining method; phylogenetic tree analysis of 343 bp fragments from 17 HEV isolates obtained in the studied population and standard genotypes from NCBI database.

All 17 HEV isolates belonged to genotype 1 with >98% sequence homology.  Figure 1 shows the phylogenetic tree constructed for the 17 new sequences and their closest matching sequences in GenBank. To further classify the subtype, we included some well-characterized subtypes for phylogenetic analysis.

Figure 1 shows that among the 17 (6 AVH with accession nos. FJ231474, FJ231475, FJ231477, FJ231480, FJ231481, FJ231482; 7 ALF with accession nos. FJ231472, FJ231473, FJ231476, FJ231483, FJ231484, FJ231485, FJ231486; remaining 4 the accession nos. belong to ACLF) HEV isolates 12 belonged to genotype 1a and 5 belonged to genotype 1c. The sequences of these 17 HEV isolates shared 91.3%–98.5% sequence homology among themselves. All AVH and ACLF isolates belonged to genotype 1a and two ALF isolates belonged to genotype 1a and the remaining five were clustered with genotype 1c. HEV isolates which belonged to genotype 1c had 90.1%–93.5% sequence homology with subtype 1c and HEV isolates which belonged to genotype 1a had 91.5%–94.2% sequence homology with subtype 1a. There was a high degree of variability found between genotypes 3 and 4, with a divergence of 31.2% and 30.1%, respectively.

Patients with genotype 1c had significantly higher peak alanine aminotransferase levels (median 2930 IU/L, interquartile range 1837–3763 versus 1324 IU/L, 945–2317; *P* = 0.01) than genotype 1a and the prothrombin time was lower in the genotype 1c patients (61%, 42–77 versus 84%, 70–96; *P* = 0.05).

## 5. Discussion

Up to now, divergent HEV sequences have been reported from many countries and fall into 4 major genotypes, namely, genotypes 1, 2, 3, and 4 [8]. Based upon nucleotide differences, it is proposed that five, two, ten, and seven subtypes for HEV genotypes 1, 2, 3, and 4 designated respectively [9]. However, inspite of the genomic diversity of HEV, studies conducted so far [20, 21] indicate that all strains identified till date comprise a single serotype [9, 22]. 

It is reported that genotypes 1 and 4 continue to circulate in humans and pigs, respectively, from India [23, 24]. However, identification of two different isolates from the same region of Japan indicated that there could be significant diversity between strains from the same region [25]. Subsequently diversity was found between various isolates from Argentina, Austria [26, 27], Spain [28], China, and Taiwan [29, 30]. 

Growing reports suggest that different HEV genotypes and subtypes play important role in disease severity. Genotypes 1 and 2 have similar epidemiological and sporadic features and can result in acute hepatitis, acute liver failure, and acute-on-chronic liver failure while genotypes 3 and 4 were normally considered to cause acute, self-limiting illness followed by a complete recovery. Thus in human genotypes 3 and 4 seem to be less virulent than genotypes 1 and 2 [31] and do not cause severe liver diseases [32]. HEV genotypes may affect the effectiveness of viral transmission and, in turn, the severity of HEV, associated hepatitis; therefore we classified patients into three groups on the bases of disease severity. 

Genotype 1 of human HEV is most prevalent in Asian countries. Nevertheless, several genotypes circulate in the neighbouring countries, such as China, Thailand, or Vietnam, where genotypes 3 and 4 have also been identified from human and swine infections. Studies demonstrated that human and swine HEVs from Western and Southern India belong to different genotypes; genotype 1 circulates in humans whereas genotype 4 in pigs [33–35] and the majority of the Indian isolates belong to subtypes 1a, 1b, 1c, and 1d, though HEV genotypes appear to be segregated geographically [36]. Single Ind-FHF isolates were studied to form genotype ID. Although all other Indian isolates were from either western or southern parts of India, the exact location of isolation of isolate Ind-FHF is not known [36]. In a recent report from North India subtypes 1a and 1c are prevalent with the subtype 1c showing a trend towards fulminancy [37]. This diversity of genotypes and subtypes is useful in understanding epidemiological phenomena such as geographical spread of the virus or the transmission in the community.

In the present study, the strains phylogenetically clustered into genotype 1 and formed two subgroups “a” and “c,” sharing 91.5%–94.2% and 90.1%–93.5% homology with intersubgroup and 79.5–85.3%, 80.1–84.8%, and 78.3–80% intrasubgroup identity homology with subgroup “b”, “d,” and “e,” respectively. This suggests that in Northwest India, different subgroups may be present. Interestingly, we also observed that the patients with genotype 1c tend to have more clinical manifestation than those with genotype 1a infection. 

## 6. Conclusion

In conclusion, the circulating genotypes and subtypes have important epidemiological implication. The present study indicates that genotype 1 is the main prevalent type of the genotypes in humans in North West India with subgroup “a” and “c.” AVH and ALF patients have different subtypes “a” and “c,” respectively, which raises our insight on the molecular basis of HEV disease severity.

## Figures and Tables

**Table 1:** Characteristic features of the patients.

Parameter	AVH	ALF	SAHF
No. of cases	100	75	30
Male : female	63 : 37	54 : 21	21 : 9
No. of pregnant females	11	16	0
Age (yrs)	23.1 ± 4.65	24.67 ± 3.37	27.75 ± 9.31
Height (cms)	151.43 ± 5.06	150.30 ± 4.23	154.92 ± 4.75
Weight (Kg)	51.05 ± 5.76	46.69 ± 4.36	51.61 ± 6
PT (seconds)	15.1 ± 1.9	29.8 ± 12.3	14 ± 0.81

PT: prothrombin time.

Data expressed as mean ± SD (range).

**Table 2:** Clinical features of the patients.

Parameter	AVH (100)	ALF (75)	SAHF (30)
Fever	42.8	77.7	66.6
Pruritus	66.6	22.2	33.3
Anorexia	57.1	66.6	100
Pedal edema	0.0	77.7	68.2
Ascites	63.3	72.1	67.3

Data expressed as percentage (%).

**Table 3:** Different biochemical parameters.

Parameter	AVH	ALF	SAHF	*P* value
Hb (g%)	28 ± 10.4	34.5 ± 9.7	37.5 ± 14.5	0.013
AST (IU/mL)	394.8 ± 440.6	905.9 ± 590.3	584.2 ± 644.8	0.000
ALT (IU/mL)	540.1 ± 551.9	1067.8 ± 642	749.2 ± 750.8	0.035
S. bil (mg/dL)	6.07 ± 4.2	9.3 ± 5.4	6.5 ± 4	0.060
ALP (IU/mL)	751.1 ± 612.8	1272.2 ± 710.6	822.5 ± 750.1	0.000

Data expressed as mean ± SD.

*P* value <0.05 consider as statistically significant.
